# Segmental range of motion of vertebral body tethering: an in-vitro analysis of single-tether, double-tether, and hybrid constructs

**DOI:** 10.1186/s13018-025-06455-y

**Published:** 2025-11-19

**Authors:** Marx Ribeiro, Eduardo A. Fancello, Jana Seggewiß, Johannes Greven, Andreas Prescher, Bernd Markert, Marcus Stoffel, Agnes Beckmann, Stephanie Da Paz, Per D. Trobisch, Miguel Pishnamaz, Frank Hildebrand, Philipp Kobbe, Jörg Eschweiler, Luis F. Nicolini

**Affiliations:** 1 Department for Trauma and Reconstructive Surgery, University Hospital Halle (Saale), Ernst-Grube-Straße 40, 06120 Halle (Saale), Germany; 2 Department of Mechanical Engineering, Federal University of Santa Catarina, R. Lauro Linhares 1850, Florianópolis, 88070-260 Brazil; 3 Department for Trauma and Reconstructive Surgery, BG Hospital Bergmannstrost Halle (Saale), Merseburger Str. 165, 06112 Halle (Saale), Germany; 4 Department for Orthopedics, Trauma and Reconstructive Surgery, University Hospital RWTH Aachen, Pauwelsstraße 30, 52074 Aachen, Germany; 5 Department of Anesthesiology and Intensive Care, University Hospital of Cologne, Kerpener Str. 62, 50937 Cologne, Germany; 6 Department for Thorax Surgery, University Hospital RWTH Aachen, Pauwelsstraße 30, 52074 Aachen, Germany; 7 Institute of Molecular and Cellular Anatomy, University Hospital RWTH Aachen, Pauwelsstraße 30, 52074 Aachen, Germany; 8 Institute of General Mechanics (IAM), RWTH Aachen University, Eilfschornsteinstraße 18, 52062 Aachen, Germany; 9 Department of Spinal Surgery, Eifelklinik St. Brigida, Kammerbruchstraße 8, 52152 Simmerath, Germany; 10 Department of Mechanical Engineering, Federal University of Santa Maria, Av. Roraima 1000, Santa Maria, 97105-900 Brazil

**Keywords:** Scoliosis, Vertebral body tethering, Growth modulation, Fusionless, Curve correction, Range of Motion

## Abstract

**Purpose:**

Vertebral Body Tethering (VBT) is emerging as a promising approach for treating Adolescents with Idiopathic Scoliosis. This study aims to address the limited experimental research on vertebral body tethering by examining its biomechanical effects on the segmental spinal range of motion (ROM).

**Methods:**

Six human spine samples (T10-L3) were subjected to pure moment testing under four different conditions: native, and instrumentation with single-tether (T10-L3), double-tether (T11-L3), and hybrid (T12-L2) techniques in flexion (FL) and extension (EX), lateral bending (LB), and axial rotation (AR). The intersegmental ROM was measured from sensors inserted in each vertebra using an electromagnetic tracking system.

**Results:**

All instrumented cases preserved at least 80% of the native segmental ROM during FL-EX for all tested segments. In AR, all segments preserved at least 88% ROM mobility for single-tether and double-tether, or 65% for the hybrid technique. In LB, the ROM was reduced to 55% for a single-tether, 47% for a double-tether, and 29% for a hybrid system. The hybrid construct tended to relatively increase the ROM of adjacent levels near the titanium rod when compared with the single-tether or double-tether.

**Conclusion:**

This study provided experimental data on individual segment motion under VBT. The findings indicate that VBT techniques preserve a significant portion of FL-EX and AR ROM for all segments. However, the tested VBT constructs provide stability for the spine in LB.

## Introduction

Idiopathic scoliosis (IS) is a three-dimensional (3D) spinal deformity that affects 0.5—5% of children, with adolescent IS (AIS) accounting for 90% of the cases in individuals aged 11–18 years [1]. In cases of severe scoliosis, surgery is recommended for spinal curvatures bigger than 45° [2]. Spinal fusion instrumentation is the standard surgical treatment to correct and prevent the progression of scoliosis, although this technique reduces the mobility of the spine and may accelerate adjacent segment degeneration over the years [3–6]. For this reason, if a young patient with remaining growth potential, vertebral body tethering (VBT) is an alternative for preserving both spinal growth and motion [6, 7].

The VBT approach is an innovation in surgical techniques to correct AIS. This technique uses an anterior approach, spinal instrumentation with vertebral body screws, and a cable compressing the convexity of the curve. This technique leverages the ‘Hueter-Volkmann Law’, promoting the gradual improvement of the curve over time through the growth modulation effect [8–10]. “The ‘Hueter-Volkmann Law’ proposes that growth is retarded by increased mechanical compression and accelerated by reduced loading in comparison with normal values” [11]. Several clinical studies reported positive outcomes for those who underwent VBT, demonstrating improved spinal alignment and curve correction [12–15].

However, there remains a need for further improvement of the system and a better understanding of the biomechanical effects of this system on the spine, since it may lead to problems such as over- or under-correction, tether breakage, surgical pulmonary complications, and higher revision surgery when compared with Posterior Spinal Instrumented Fusion [12, 16–19]. Furthermore, since VBT is a motion preservation technique, one of its potential advantages is minimizing adjacent-level degeneration that can occur in spinal segments adjacent to a fused area after traditional spinal fusion. This phenomenon is partially attributed to the increased stress and altered biomechanics placed on the adjacent levels following fusion [4, 5]. Moreover, long-term studies and experiences are needed to clarify if VBT can protect the intervertebral disc (IVD) from degeneration [20–22].

In addition to clinical studies, animal models have provided valuable insights into the growth-friendly potential and correction of the VBT [23–26]. They demonstrated that it is possible to create scoliosis in immature spines, thus showing the potential for altering the biomechanical behavior of the spine. Furthermore, the studies showed evidence of soft tissue preservation in the instrumented porcine and bovine spine [23–26]. However, there are limitations of animal models when translating their findings into human models due to their differences, such as material properties, duration, non-bipedal motion, and geometry [27].

To complement the investigation, there have been attempts to gain further insights into the biomechanical behavior of the spine through in vitro studies using human cadaver spines [28–30]. These studies aimed to provide a controlled and reliable laboratory setting to investigate the effects of VBT on the spine, which provides a closer representation of human anatomy and biomechanics compared to animal models, despite the usual *in-vitro* test limitations like degeneration, absence of stabilizing soft tissue, and missing homeostasis of tissue in such approaches [31, 32]. However, the literature on in vitro studies specifically focused on VBT instrumentation remains scarce. This knowledge gap highlights the need for further research in this area to enhance the understanding of the biomechanical behavior of the spine under different VBT techniques, especially regarding the potential to keep the global and segmental range of motion (ROM) in the sagittal and transverse planes [29, 30].

This study aims to investigate the segmental ROM distribution for different VBT techniques (single-tether, double-tether, and hybrid). We hypothesize that the behavior of ROM in individual segments maintains a trend like the global instrumented spine when submitted to VBT single-tether, double-tether, and hybrid techniques.

## Materials and methods

### Specimens, testing protocol, and surgical groups

**Fig. 1:**
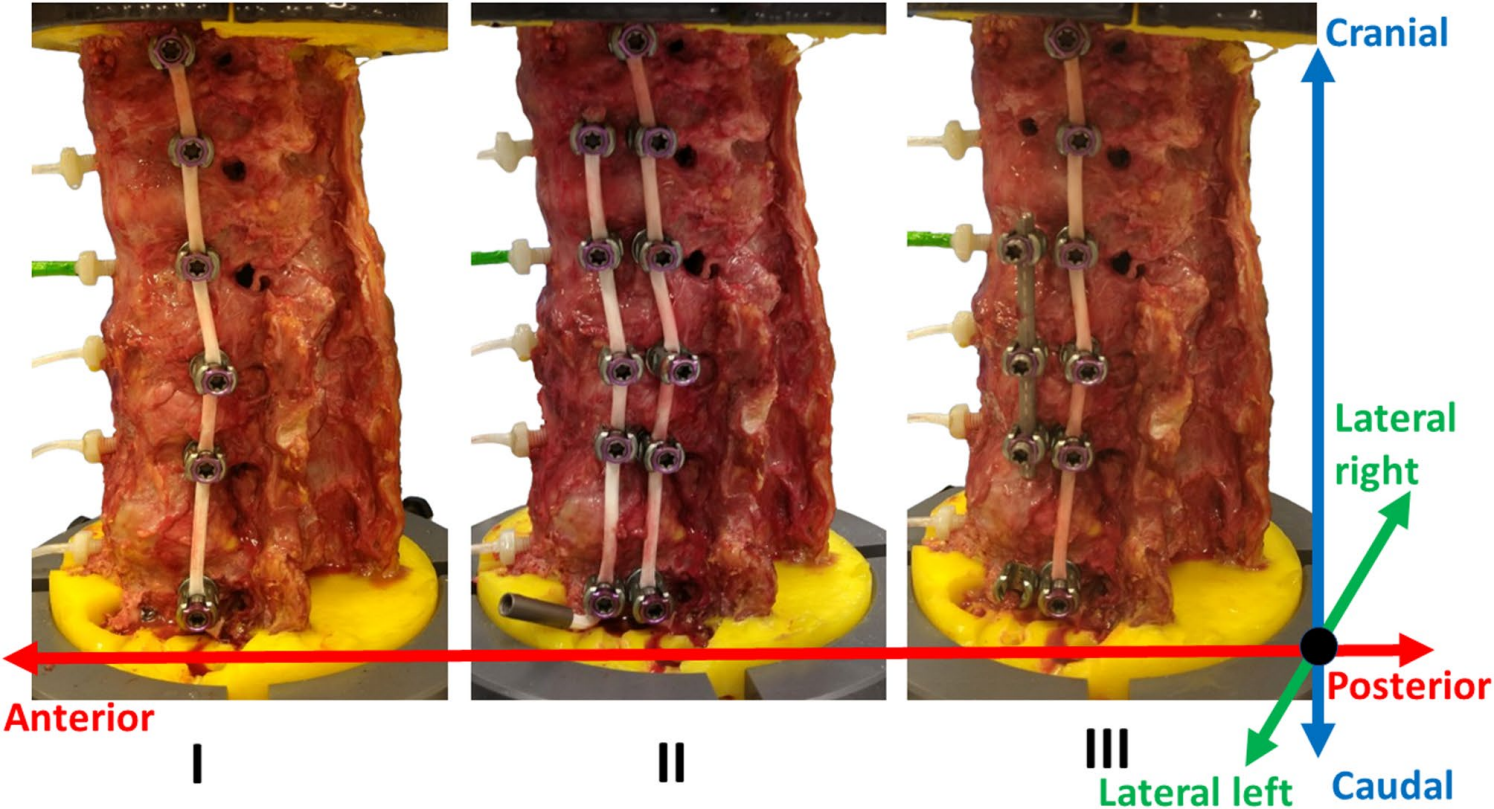
Lateral view of T10-L3 spine with (I) single-tether, (II) double-tether, and (III) hybrid construct including one tether and a titanium rod

Six fresh frozen spinal specimens T10-L3 from donors (5 female and 1 male) with a mean age of 82 years at death (73–88 years) and without spinal deformities were prepared for biomechanical flexibility tests in the three anatomical directions (flexion (FL) -extension (EX), lateral bending (LB), axial rotation (AR)) following an established testing protocol [29, 33, 34]. The study was conducted with ethical approval from the RWTH Aachen University committee (No. EK 280/20). Experiments were performed using a custom-built spine testing rig placed on a special machine (DYNA-MESS®, Stolberg, Germany) [29]. Measurements were recorded using torque transducers and an electromagnetic tracking system (EMT) (Aurora® NDI Europe GmbH, Radolfzell, Germany). The specimens were tested under native and different instrumented surgical VBT techniques: a single-tether spanning all vertebrae from T10 to L3 with pre-tension of 100N [28–30], a double-tether as an additional cord from T11 to L3 without pre-tension, and hybrid instrumentation with the single tether and a titanium alloy rod from T12 to L2 (Fig. 1), all instrumented cases on the left side of the spine. The tether pre-tensioning allows immediate correction of the spinal deformity by tending to compress the convex side and stretch the concave side. The increase of pre-tensioning can significantly reduce spinal motion [35] but is relevant for correcting curve deformity, particularly in severe or stiff curves. Meanwhile, excessive pre-tension in the clinical scenario increases the risk of tether failure and overcorrection, with the maximum tether pre-tension load ranging from 300 to 400 N [12]. The selected pre-tension of 100 N reflected a commonly used clinical value and has been adopted in previous cadaveric studies [28, 30]. This level was also chosen to avoid excessive scoliotic angle and screw pullout, thereby complicating the testing procedure with the described experimental setup. The second tether was left unloaded as a reinforcement, or “safety cable,” to reduce stress on the primary tether and, therefore, lower the risk of breakage or loss of correction. It was intentionally left unloaded to avoid contributing to initial curve correction or altering the neutral spinal position. Additionally, this strategy helps preserve spinal motion while providing stability in the event of primary tether failure.

The instrumentation was provided by Globus Medical Inc. (Audubon, PA, USA). The tests were performed under a pure moment load of up to ± 6 Nm for three cycles at a speed rate of 1°/s [28]. The last cycle was considered for evaluation. The testing sequence was randomized for both the VBT technique and load direction.

This study presents a comprehensive ROM analysis for individual segments from T10 to L3, extending upon our previously reported experimental findings in Nicolini et al. [29]. In our prior work, we focused on the global ROM and analyzed the specific L1-L2 segment. The current study broadens the scope by performing an in-depth ROM analysis for each segment within T10-L3, including L1-L2 data [29]. Furthermore, we introduce assessments of the T11-L3 and T12-L2 regions, which are influenced by double tether and hybrid instrumentation, respectively, but were not previously investigated.

### Data analysis

The position and orientation of each segment were tracked by an EMT system for each segment. The sensors were implanted in the center of the segment’s body. A surgical expert realized this, and an engineer supported the intervention. The six degrees of freedom (6 DOF) data were recorded as Cartesian Coordinates for translational motion and Euler angles for the rotational movement, respectively. The angular displacement between two vertebral segment bodies (segmental ROM) was calculated using the method described in Beckmann et al. [33, 34] where the relative angle between two vertebrae was projected as a 2D-angle in the plane perpendicular to the pure moment applied [29, 36]. The middle curve of the hysteresis in the third cycle was used to evaluate the 2D-ROM. Data were excluded from the analysis when defect sensors or gimbal locking in the Euler angles were present.

The statistical analysis was performed with a script in Python 3.9.12 with the SciPy library [37]. Sphericity and non-normal distribution were tested with Mauchly’s sphericity test and the Shapiro–Wilk test, respectively. Next, a one-tailed Wilcoxon signed-rank test for repeated measures was applied to determine the significance between the groups in case of a non-normal distribution. A significant level of 0.05 was considered for statistical analysis (*p* < 0.05). Medians with interquartile range were evaluated. The reductions were observed in the following order: instrumented with one tether, two tethers, and hybrid construction. Utilizing median ROM values, we provide a reliable measure of central tendency for our observations, capturing the typical ROM within each condition. Unlike the mean, which is influenced by extreme values, the median provides a robust measure of central tendency, particularly in datasets with outliers or skewed distributions, providing a clearer understanding of spine behavior under various instrumentation combinations.

## Results

### Range of motion of the thoracolumbar segments

**Fig. 2:**
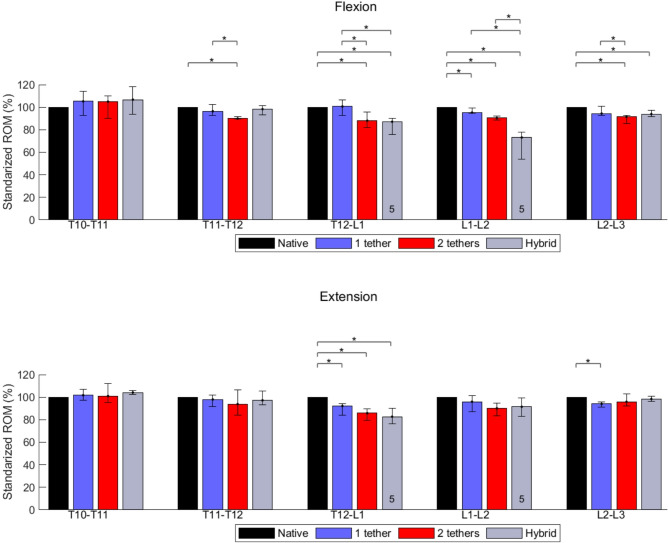
Effects of different surgical constructs on the ROM of the spine in flexion and extension. The data were standardized concerning the ROM of the native spine. The bars and error bars represent the medians of 2D ROM and interquartile ranges, respectively. The symbol * represents *p* ≤ 0.05. The numbers at the bottom of the bars indicate the numbers of specimens used for calculating the medians when they differ by 6 specimens

**Fig. 3:**
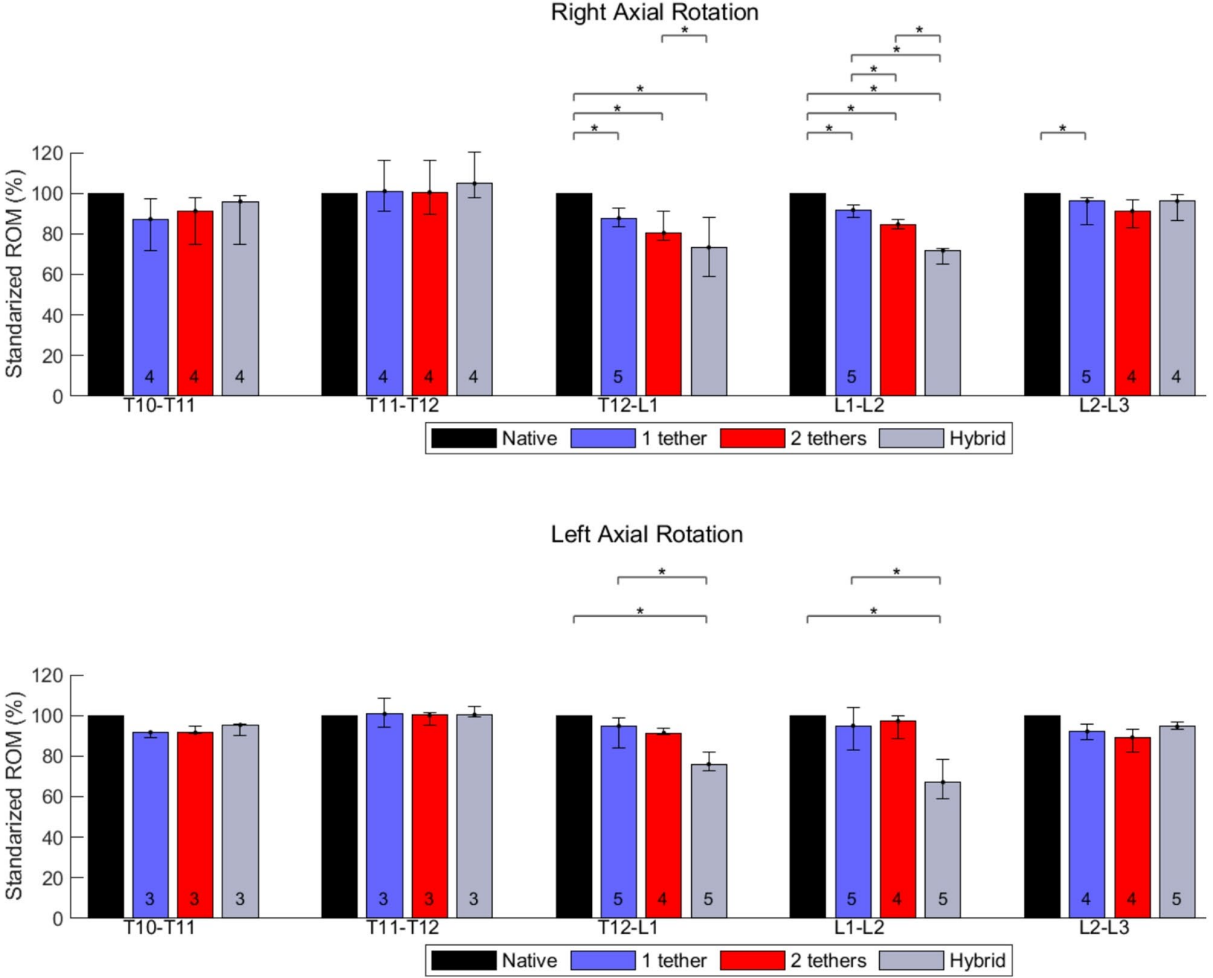
Effects of different surgical constructs on the ROM of the spine in axial rotation. The data were standardized concerning the ROM of the native spine. The bars and error bars represent the medians of 2D ROM and interquartile ranges, respectively. The symbol * represents *p* ≤ 0.05. The numbers at the bottom of the bars indicate the numbers of specimens used for calculating the medians when they differ by 6 specimens

**Fig. 4:**
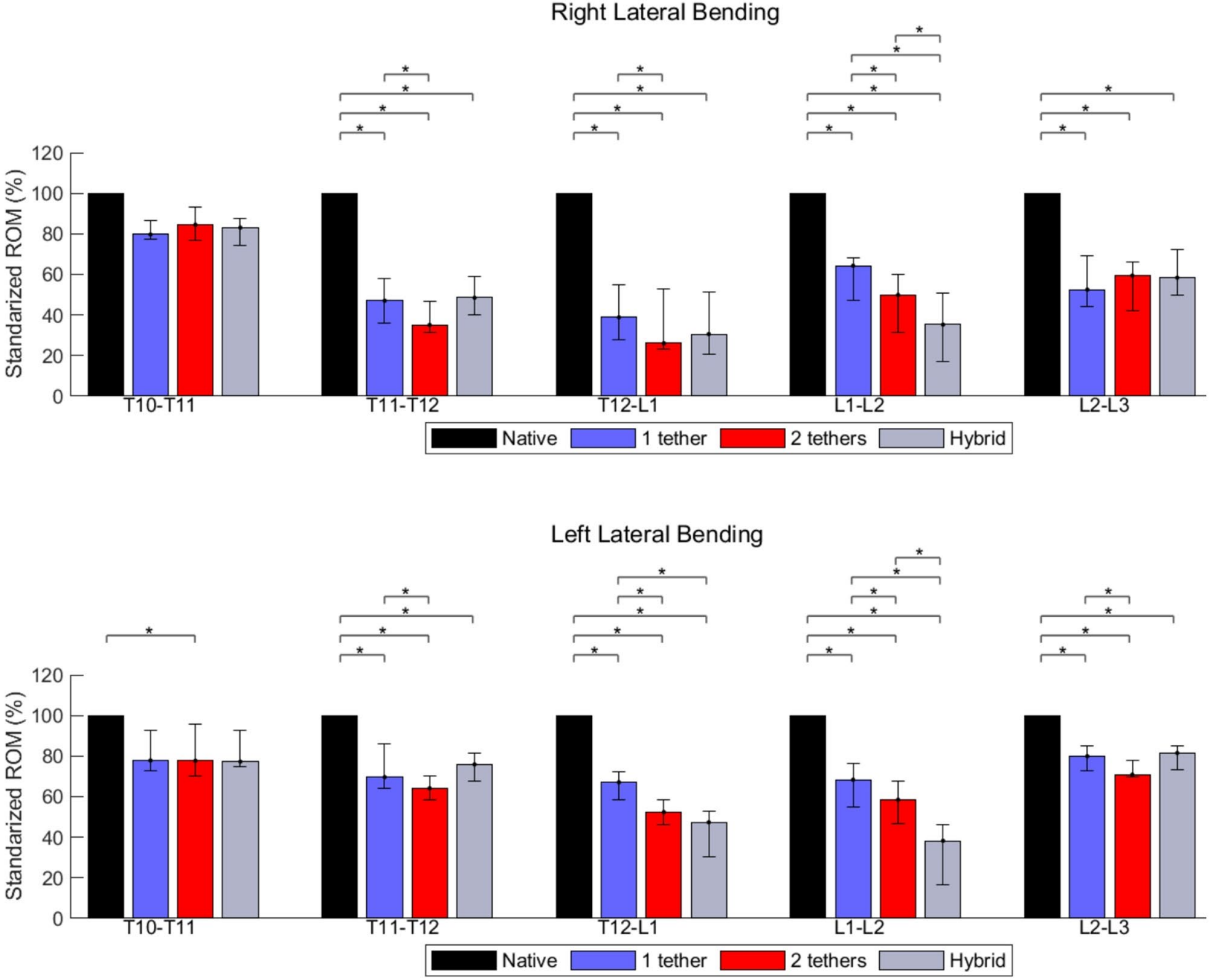
Effects of different surgical constructs on the ROM of the spine in lateral bending. The data were standardized concerning the ROM of the native spine. The bars and error bars represent the medians of 2D ROM and interquartile ranges, respectively. The symbol * represents *p* ≤ 0.05. The numbers at the bottom of the bars indicate the numbers of specimens used for calculating the medians when they differ by 6 specimens

**Fig. 5:**
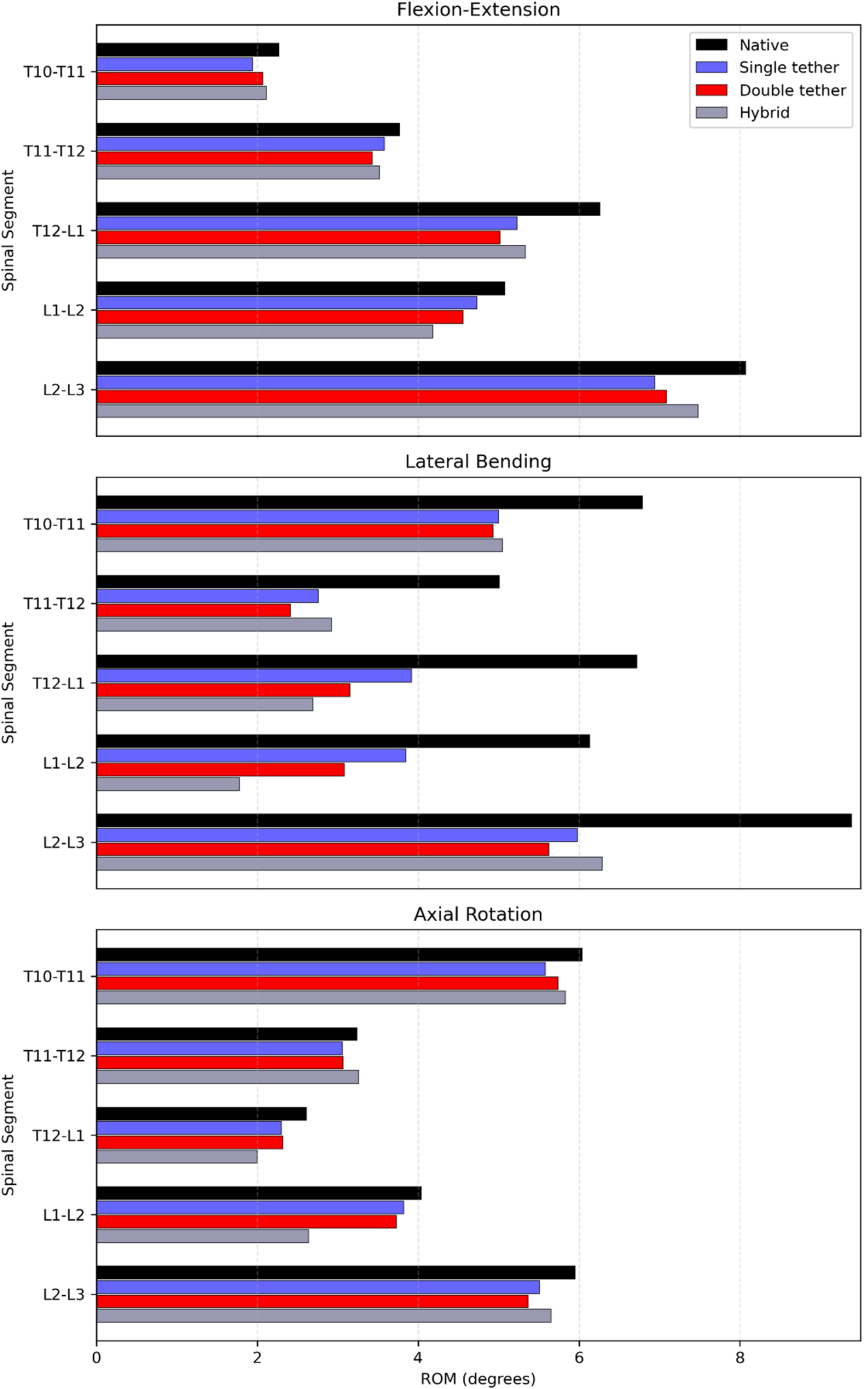
Effects of different surgical constructs on the ROM of the spine in FL-EX, LB, and AR. The data represent the medians of the 2D-ROM in degrees

The medians of the ROM of individual segments are presented in Table 1. The statistical analysis for significant changes is shown in Figs. 2, 3, and 4, and complemented by Fig. 5.

**Table 1:** Medians of the segmental ROM (°) of the native and instrumented spine instrumentation with single-tether (T10-L3), double-tether (T11-L3), and hybrid (T12-L2) techniques at 6 Nm

Movement	ROM (T10-T11)				ROM (T11-T12)				ROM (T12-L1)			
	Native	Single-tether	Double-tether	Hybrid	Native	Single-tether	Double-tether	Hybrid	Native	Single-tether	Double-tether	Hybrid
Flexion	1.19	0.81	0.95	0.97	2.28	2.13	2.05	2.12	2.75	2.54	2.31	2.41
Extension	1.08	1.13	1.12	1.14	1.49	1.45	1.38	1.40	3.51	2.69	2.71	2.92
Right lateral bending	3.40	2.64	2.62	2.63	2.45	1.03	0.85	1.06	3.19	1.32	1.15	1.27
Left lateral bending	3.39	2.36	2.31	2.42	2.56	1.73	1.56	1.86	3.53	2.60	2.00	1.42
Right axial rotation	2.62	2.50	2.54	2.56	1.66	1.55	1.57	1.67	1.27	1.01	1.07	0.96
Left axial rotation	3.42	3.08	3.20	3.27	1.58	1.51	1.50	1.59	1.34	1.29	1.25	1.04
Flexion–Extension	2.27	1.94	2.07	2.11	3.77	3.58	3.43	3.52	6.26	5.23	5.02	5.33
Lateral Bending	6.79	5.00	4.93	5.05	5.01	2.76	2.41	2.92	6.72	3.92	3.15	2.69
Axial Rotation	6.04	5.58	5.74	5.83	3.24	3.06	3.07	3.26	2.61	2.30	2.32	2.00

Movement	ROM (L1-L2)				ROM (L2-L3)			
	Native	Single-tether	Double-tether	Hybrid	Native	Single-tether	Double-tether	Hybrid
Flexion	2.50	2.37	2.14	1.83	4.86	4.02	4.14	4.32
Extension	2.58	2.36	2.42	2.35	3.21	2.92	2.95	3.16
Right lateral bending	2.54	1.56	1.11	0.68	3.86	1.64	1.72	1.80
Left lateral bending	3.59	2.29	1.97	1.10	5.53	4.34	3.91	4.49
Right axial rotation	1.67	1.51	1.42	1.07	2.94	2.82	2.77	2.79
Left axial rotation	2.37	2.31	2.31	1.57	3.01	2.69	2.60	2.86
Flexion–Extension	5.08	4.73	4.56	4.18	8.07	6.94	7.09	7.48
Lateral Bending	6.13	3.85	3.08	1.78	9.39	5.98	5.63	6.29
Axial Rotation	4.04	3.82	3.73	2.64	5.95	5.51	5.37	5.65

Data of L1-L2 partially published in [29]

During FL-EX, the T10-T11 and T11-T12 segments retained at least 85% of the native ROM for all instrumented conditions (single-tether, double-tether, and hybrid). Similarly, the T12-L1 segment, in the same cases, preserved at least 77% of the ROM of the native spine. For the lumbar segments, L1-L2 and L2-L3, the FL-EX were at least 83% of the native ROM for the single-tether and double-tether, while for the hybrid instrumentation case, the values were 73% and 89% for L1-L2 and L2-L3 segments (Table 1).

For AR, the segments T10-T11 and T11-T12 kept at least 92% of the native absolute ROM for all the instrumented cases. The T12-L1 segment preserved 88% of the native spine ROM for the single-tether and double-tether instrumentation cases, and 76% for the hybrid case. For the lumbar segments, L1-L2 and L2-L3, at least 90% of the ROM in AR was kept for single-tether and double-tether instrumentation. In the hybrid instrumentation, 65% and 95% of the native ROM in AR were preserved for L1-L2 and L2-L3 (Table 1).

The T10-T11 absolute ROM after instrumentation with single-tether, double-tether, and hybrid, kept at least 77% of the native ROM in the right LB and 68% in the left LB (Table 1). For all the instrumented cases, the absolute T11-T12 ROM ranged between 35 and 43% during right LB, and 61% and 73% for left LB direction compared to the native state. A similar trend was found in the T12-L1 segment in the right LB with a ROM reduced to values between 36 and 41%. While left LB, the T12-L1 ROM of the native spine (100%) was reduced to 74%, 57%, and 40% for single-tether, double-tether, and hybrid instrumentation (Table 1).

The highest ROM reduction in left LB happened in the L1-L2 segment, which presented a ROM of 64%, 55%, and 31% for single-tether, double-tether, and hybrid instrumentation, respectively, compared to the native state. For the same order in the right LB, the ROM was 61%, 44%, and 27%. The ROM of the L2-L3 segment during right LB was reduced to 42%, 43%, and 47% for single-tether, double-tether, and hybrid constructs, respectively. For the same respective cases in left LB, the ROM reduced to 79%, 71%, and 81% (Table 1).

### Thoracolumbar segments spanned by single and double tether (T11-L3)

**Fig. 6:**
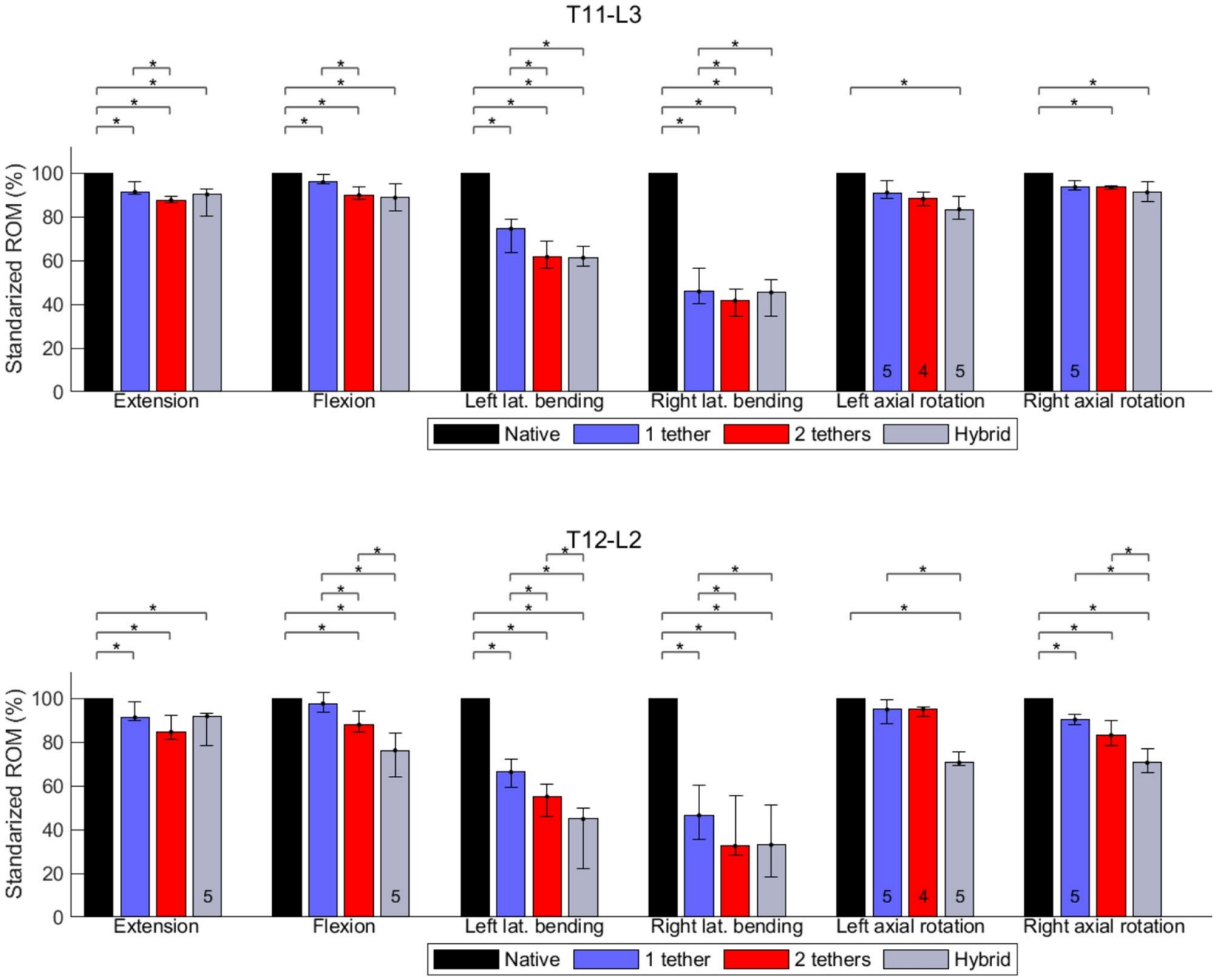
Effects of different surgical constructs on the ROM of the spine in FL-EX, LB, and AR. The data were standardized concerning the ROM of the native spine. The bars and error bars represent the medians of 2D ROM and interquartile ranges, respectively. The symbol * represents *p* ≤ 0.05. The numbers at the bottom of the bars indicate the numbers of specimens used for calculating the medians when they differ by 6 specimens

During testing, the segments T11-L3 were completely spanned by the single and double tether construct. Data of T12-L2 was presented from [36]. For all construct combinations, the instrumented spine segments kept at least 90% of the native ROM during FL or EX (Table 2). Significant reductions were found between native and all instrumented cases (Fig. 6). Additionally, a significant difference in ROM between single- and double-tether was found.

**Table 2:** Medians of the ROM (°) of the native and instrumented spine instrumentation with single-tether (T10-L3), double-tether (T11-L3), and hybrid (T12-L2) techniques at 6 Nm

Movement	ROM (T11-L3)				ROM (T12-L2)			
	Native	Single tether	Double tether	Hybrid	Native	Single tether	Double tether	Hybrid
Flexion	13.73	13.12	12.35	12.34	5.25	4.91	4.45	4.24
Extension	11.90	11.21	11.16	11.26	6.02	5.05	5.14	5.27
Right lateral bending	11.31	6.18	5.67	5.35	5.73	2.87	2.26	1.94
Left lateral bending	15.49	11.50	10.43	10.28	7.12	4.89	3.98	2.52
Right axial rotation	9.51	8.91	8.45	8.09	2.94	2.52	2.49	2.03
Left axial rotation	8.31	7.69	7.60	7.24	3.72	3.60	3.55	2.61
Flexion–Extension	25.63	24.33	23.51	23.60	11.27	9.96	9.59	9.51
Lateral Bending	26.80	17.68	16.10	15.63	12.85	7.76	6.24	4.46
Axial Rotation	17.82	16.60	16.05	15.33	6.66	6.12	6.04	4.64

Considering the behavior of T11-L3 (Table 2), the instrumented spine preserved at least 85% of the native ROM in AR. Significant ROM reductions were found between the native spine and instrumented spine with double-tether in right AR and between the native spine and hybrid construct for both left and right AR. During LB, ROM reductions of 34%, 40%, and 42% happened, respectively, for single-tether, double-tether, and hybrid instrumentation in comparison to the native state. All ROM comparisons had statistical significance (Fig. 6), for both left LB (towards the tether) and right LB (away from the tether), with the only exception being between double-tether and hybrid constructs.

### Thoracolumbar segments spanned by hybrid construct (T12-L2)

For testing the hybrid construct, the segments T12-L2 were instrumented with a titanium rod. During FL-EX, the hybrid construct kept 84% of the native ROM. During AR, the instrumentation with one or two tethers preserved at least 91% of the native AR, while the hybrid construct kept 70% of its native ROM (Table 2). During LB, reductions of 40%, 51%, and 65% in ROM occurred after inserting the single-tether, double-tether, and hybrid construct, respectively. For the same instrumented sequence, ROM reductions of 31%, 44%, and 65% occurred during left LB, while in right LB the reductions were 50%, 60%, and 66%. These reductions were significant for most of the groups (Fig. 6).

## Discussion

VBT is a relatively new scoliosis treatment technique with promising outcomes. Despite many achievements obtained with experimental studies with animal models investigating the effect of VBT [22–24, 33, 34, 36], few biomechanical studies with the human spine were conducted [28–30]. Moreover, these studies focused on the investigation of the global ROM of the spine. This study is the first to investigate the effects of different VBT constructs on the segmental ROM of the thoracolumbar spine (T10-L3).

Across FL-EX and AR, VBT constructs preserved ≥ 80–92% of the motion of the native spine at the segmental level. Single-tether and double-tether constructs maintained mobility throughout T10–L3, while the hybrid system maintained mobility in adjacent segments but, as expected, reduced motion at fused levels (T12–L2). These results align with prior global ROM reports [28–30], and highlight the motion-preserving superiority of VBT compared with fusion.

For the T11-L3, the statistical tests showed significant ROM reduction between native and all instrumentation constructs, and significant reduction between single-tether and double-tether, and the hybrid system. Additionally, the double-tether and hybrid constructs were equivalent in our study (*p* = 0.218 for left LB, *p* = 0.781 for right LB). Two factors can contribute to this finding: one is the extra stiffness provided by the second tether, and the other is that the hybrid system is not completely fused, allowing some rotation in the rod fixation screws. Although this global equivalence, the segmental analysis of the hybrid instrumentation induced a ROM increase in LB for the segments adjacent (T11-T12, L2-L3) to the fused ones with the titanium rod (T12-L2). It can be concluded that the adjacent segments to the titanium rod of the hybrid system are not protected against overloading. Although the ROM increase might be the result of increasing specimen decomposition, additional tests covering the biomechanical behavior of the disc and the segmental ROM of adjacent levels are still needed. These findings raise concerns of adjacent segment degeneration in the case of the hybrid system and potential adjacent preservation with the double-tether technique.

The investigation of the biomechanical outcomes of VBT, particularly its hybrid system, is still in its beginning, lacking comprehensive long-term follow-up data. This requires a cautious approach to interpreting current findings, especially where the trend of increased adjacent mobility is shown. However, the risk of adjacent segment degeneration with hybrid VBT is reasonable in light of the extensive fusion literature. In this context, the work of Lonner et al. [5] serves as a significant reference point. Their study encompassed a 10 years follow-up of AIS patients who underwent spinal fusion, indicating a 7.3% incidence of disc degeneration. This degeneration was predominantly observed in the caudal segments adjacent to the lower instrumented vertebra (LIV), specifically at LIV + 2 and LIV + 3 levels. Moreover, their findings suggest a progressive trend in such degeneration. Indeed, this trend was found in a recent publication by Burrows et al. [4] who carried out a 21 years follow-up study for AIS patients who underwent spinal fusion surgery. The authors found that 64% of the patients had evidence of adjacent segment degeneration [4]. These observations underscore the necessity for long-term studies on VBT to comprehensively evaluate and contrast its biomechanical impacts, particularly on adjacent segment degeneration, against those observed with traditional fusion techniques in AIS treatment.

Evidence on disc health after VBT is still mixed. The authors Yucekul et al. and Hoernschemeyer et al. found no evidence of disc degeneration in patients who underwent VBT surgery [20, 21]. Conversely, Jackson et al. found a significant (*p* = 0.0075) increase in Pfirrmann disc grade in the discs spanned by the tethering, but not in the adjacent levels [22]. Taken together, these studies suggest that while VBT may not accelerate adjacent degeneration within the first three years post-surgery [20–22]. However, further investigation of the VBT effect on the disc’s health is necessacry. Although Hoernschemeyer et al. [20] did not find evidence to support disc degeneration, they found that 22% (2/9) of the patients developed facet osteoarthritis in the lower lumbar spine, in addition to the 44% of patients who already presented this pathology previously to the VBT surgery. These findings stress the need for further research on the preservation capacity of VBT, particularly for hybrid constructs that combine tethering and fusion.

Motion preservation is especially relevant in the lower thoracic and lumbar segments, given their higher flexibility and physiological load compared to the upper thoracic segments. The thoracolumbar region is reported to have a higher incidence of tether breakage within VBT [17, 38]. Baroncini et al. [39] showed that the time of the breakage affects the correction outcome. Aiming to reduce early tether breakage, double-tether, and hybrid systems started to be used by surgeons [38]. For this reason, the present study focused on investigating the biomechanics of the thoracolumbar spine instrumented with not only a single-tether, as one would expect, but also a double-tether and hybrid system. Trobisch et al. [40] found that, after a two-year follow-up, a suspected tether breakage rate occurred in 90% of the patients. However, despite this breakage rate, a lumbar curve correction of 50% was achieved, reinforcing the importance of the timing of the tether breakage [39]. Additionally, the shown tendency of keeping the thoracolumbar mobility follows the in-vivo flexibility studies where after 1-year follow-up the lateral bending mobility was reduced by 54% in the lumbar [41] and 77% in the thoracic spine when compared with preoperative flexibility, while the movement was kept steady for FL-EX [41, 42].

In summary, VBT constructs preserved the majority of motion across segments, particularly in FL-EX and AR, whereas hybrid constructs limited motion at fused levels but showed a tendency to increase it in adjacent segments. These observed motion preservation findings, especially in FL-EX and AR, may directly correlate with the clinical outcomes as better patient life quality and satisfaction, in addition to disc preservation [20–22, 43, 44].

Although this study focused on the thoracolumbar spine, VBT originally emerged as a growth-friendly surgery for thoracic curves, which represent the majority of AIS cases. Therefore, it is important to highlight biomechanical differences between the thoracic and thoracolumbar regions. The thoracic spine—characterized by smaller disc heights in the mid-thoracic segments—typically exhibits reduced flexibility in FL-EX and LB compared with the lumbar region. In addition, the rib cage and sternum reinforce the thoracic spine and further restrict AR [45]. Moreover, the rib cage increases thoracic spinal stability in all motion planes, but predominantly in the upper thorax half [46]. By stabilizing the spine, the rib cage reduces both ROM and probably corrective potential but simultaneously decreases implant stresses, which may prolong construct longevity. This might explain that the risk of tether rupture is significantly larger in the lumbar spine compared to the thoracic region when using VBT [17, 40].

Aged spines tend to change the sagittal alignment, which can exhibit progressive reduction in lumbar lordosis and increased thoracolumbar kyphosis patterns, well-documented in aging populations [47–49], and are pertinent to our study, given the elevated donor ages. Biomechanically, such baseline kyphosis can shift the neutral posture, thereby altering both the absolute FL-EX ROM and the new post-instrumentation neutral position.

Interestingly, sagittal malalignment is not uncommon in AIS. Abelin-Genevois et al. and Schlösser et al. [50, 51] reported pathological sagittal profiles in 41% of mild and 56% of severe AIS cases. Among severe cases, thoracic hypokyphosis (Type 2a) occurred in 39%, whereas thoracolumbar kyphosis (Type 2b) appeared in 8%. Baroncini et al. [52] investigated sagittal alignment in AIS patients undergoing VBT surgery and found that 55% of the cases show pathological malalignment, with 16% classified as Type 2b sagittal profile. After 2 years, VBT had a positive influence on sagittal parameters and did not induce lumbar kyphosis.

The limitations of this study are the specimens obtained by body donors which were healthy spines but in advanced age, which holds obvious differences to translation to a teenage scoliotic spine, such as bone quality, disc properties, tissue elasticity, and age-related degenerative changes, in addition to the *post-mortem* tissue degradation inherent to cadaveric studies, which may alter the biomechanical response under load and influence implant behavior. The boundary conditions and simplified loading scenarios applied in laboratory settings do not reflect the dynamic and multifactorial forces acting on the spine during real-life activities, which also limits the direct translation to the clinical scenario. The limited number of specimens (n = 6) affects the statistical power of this study and the more precise population representation. Moreover, the technical failure of some performed tests further reduces the power of this study. Future studies with a larger sample size are needed to validate the findings. The tests were performed with no variation of pre-tension in the tether. Additionally, since the specimens’ instrumentation was completely spanned with at least a single tether, no segmental ROM analysis was performed on the adjacent segments to the single-tether system. These limitations could be overcome by, if feasible, using young scoliotic specimens representing potential patients for VBT treatment. A physiological scenario could be potentially achieved by applying a compressive follower load [53], and using a bioreactor to maintain a moist and body temperature environment [33]. Moreover, the spine can be tested with different speed protocols to understand its dynamic behavior under various VBT configurations.

The study aimed to provide fundamental biomechanical data on the immediate mechanical effects of VBT constructs on cadaveric healthy, mature spinal segments, under laboratory-controlled conditions. One limitation of this study is that the application of a pure moment may not adequately represent the complex physiological loads experienced in vivo. The study does not account for the complex loading patterns that occur during functional activities. However, the application of pure moment without axial loads is widely used as a protocol for spinal tests, while it provides uniform loading across all spinal levels, is relatively easy to reproduce, and may produce forces and moments in implants comparable with loads observed in vivo [54, 55]. Moreover, the use of 2D-angle projections may oversimplify the complex 3D motion patterns of the spine, particularly in a condition like scoliosis, which is inherently three-dimensional. However, the 2D-angle projection method is a standard and reliable approach in in vitro biomechanical testing, effectively capturing the dominant rotational motion in the plane of loading. However, given the aforementioned limitations, this study provides a baseline understanding of motion preservation characteristics, which is a prerequisite for understanding their behavior in more complex scoliotic or growing spines.

## Conclusion

This study enhances understanding of the biomechanical impact of the VBT systems, including single-tether, double-tether, or hybrid constructs, on the individual segmental ROM of the thoracolumbar spine. The results indicate that VBT techniques preserve a significant portion of FL-EX and AR ROM for all segments like the global trend, except for the hybrid system. The hybrid system reduces ROM in directly connected segments but does not reduce adjacent to the fused segments when compared with the double-tether. The double-tether system presents a smoother segmental mobility distribution along the spine than the hybrid system.

Further studies focusing on VBT instrumentation would contribute to a more comprehensive understanding of its effects on the spine, allowing for improved surgical techniques, better patient selection, and optimized treatment outcomes.

## Data Availability

All the datasets analyzed or generated during this study will be available from the corresponding author upon reasonable request.
